# Three uncommon adrenal incidentalomas: a 13-year surgical pathology review

**DOI:** 10.1186/1477-7819-10-64

**Published:** 2012-04-27

**Authors:** Rani Kanthan, Jenna-Lynn Senger, Selliah Kanthan

**Affiliations:** 1 Department of Pathology and Laboratory Medicine, Saskatoon, Saskatchewan, Canada; 2 Department of Surgery, University of Saskatchewan, Saskatoon, Saskatchewan, Canada; 3 Room 2868 G-Wing, Royal University Hospital, 103 Hospital Drive, Saskatoon, Saskatchewan, S7N 0 W8, Canada

**Keywords:** Adrenal incidentaloma, Adrenal ganglioneuroma, Periadrenal schwannoma, Primary adrenal pleormorphic leiomyosarcoma

## Abstract

**Background:**

The discovery of adrenal incidentalomas due to the widespread use of sophisticated abdominal imaging techniques has resulted in an increasing trend of adrenal gland specimens being received in the pathology laboratory. In this context, we encountered three uncommon adrenal incidentalomas.

The aim of this manuscript is to report in detail the three index cases of adrenal incidentalomas in the context of a 13-year retrospective surgical pathology review.

**Methods:**

The three index cases were investigated and analyzed in detail with relevant review of the English literature as available in PubMed and Medline. A 13-year retrospective computer-based histopathological surgical review was conducted in our laboratory and the results were analyzed in the context of evidence-based literature on adrenal incidentalomas.

**Results:**

A total of 94 adrenal specimens from incidentalomas were identified, accounting for 0.025% of all surgical pathology cases. In all 76.6% were benign and 23.4% were malignant. A total of 53 females (56.4%) and 41 males (43.6%) aged 4 to 85 years were identified. The benign lesions included cortical adenoma (43.1%), pheochromocytoma (29.3%) and inflammation/fibrosis/hemorrhage (8.3%). Metastatic neoplasms were the most common malignant lesions (50%) followed by primary adrenocortical carcinomas (31.8%) and neuroblastoma (13.6%). These cases were discovered as adrenal incidentalomas that led to surgical exploration.

The three index cases of adrenal incidentalomas with unusual pathologies were encountered that included (a) adrenal ganglioneuroma, (b) periadrenal schwannoma and (c) primary adrenal pleomorphic leiomyosarcoma. These cases are discussed, with a literature and clinicopathological review.

**Conclusions:**

Adrenal lesions are uncommon surgical specimens in the pathology laboratory. However, higher detection rates of adrenal incidentalomas aided by the ease of laparoscopic adrenalectomy has resulted in increased adrenal surgical specimens leading to unsuspected diagnostic and management dilemmas. Accurate pathological identification of common and uncommon adrenal incidentalomas is essential for optimal patient management.

## Background

At autopsy, adrenal masses are among the most common tumors, reported in 3% of the population over 50 years of age [1,2]. Such cases are, however, rare surgical specimens sent to the pathology laboratory. Improvements in abdominal imaging with the widespread use of non-invasive techniques such as high-resolution ultrasonography, computed tomography (CT), and MRI has generated an increased awareness and interest in the ‘incidentaloma’, adrenal masses greater than 1 cm found serendipitously on radiological imaging for an unrelated cause. Common benign lesions include adrenocortical adenoma, pheochromocytoma, myelolipoma, and adrenal hyperplasia. Malignant lesions are rare, with an incidence of approximately 1 in every 4,000 adrenal tumors. Primary adrenocortical carcinoma is often associated with a high recurrence rate and poor 5-year survival. In non-selected series, metastatic lesions to the adrenal gland account for 0% to 21% of adrenal neoplasms, while primary adrenal carcinoma has been reported in 0% to 25% [3,4].

The aim of this manuscript was to review and document the trend of adrenal specimens received in our surgical pathology laboratory over a 13-year period with special emphasis on three uncommon pathologies of adrenal ‘incidentalomas’ that were encountered, including ganglioneuroma, periadrenal schwannoma, and primary pleomorphic leiomyosarcoma.

## Methods

Pathological diagnosis of three unusual lesions resulting from the analysis of adrenal incidentaloma specimens prompted a detailed review of all adrenal lesions over the past 13 years (1997 to 2010) available in the Laboratory Information System (LIS). The LIS, operational since 1996, is a robust, reliable, centralized electronic data repository, which is administered by the Saskatoon Health Region for the Department of Pathology and Laboratory Medicine in the city of Saskatoon, Canada. All surgical material was retrieved and analyzed to confirm the histomorphological diagnosis. Histopathological review and clinicopathological correlation with the recorded patient information, including sex, age, laterality, and final diagnosis, was conducted.

A complete review using the keywords ‘adrenal’ with ‘ganglioneuroma’, ‘schwannoma’, and ‘leiomyosarcoma’ was completed within the scope of the English language as available in PubMed/Medline databases. Primary references were read and analyzed, and their reference lists were used to retrieve secondary sources. PubMed’s ‘Related Articles’ feature was an additional tool in the identification of relevant articles. Additionally, a literature review on the current status of adrenal incidentalomas was undertaken.

This study was conducted with ethics approval from the University of Saskatchewan Biomedical Research Ethics Review Committee.

## Results

### The 13-year surgical pathology review

The surgical pathology review of 381,200 cases over the 13-year period (1997 to 2010) retrieved 94 adrenal lesions (incidence of 0.025%). Patient ages ranged from 4 to 85 years, with a mean of 51.6 years (median 55 years). The majority (54%) were between the ages 40 to 60. Fine-needle aspiration specimens were rare (4/94). Females (56.4%) had a slightly higher preponderance than males (43.6%). There was a slight predominance for left-sided lesions, with 55 (58.5%) pathologies detected in the left adrenal gland and 39 (41.5%) in the right in our series. Over three-quarters (76.6%) of the pathologies diagnosed were benign (Table 1), and the remaining 23.4% were malignant (Table 2). The most common benign lesions included cortical adenomas (31 cases, 43.1% of benign lesions, 33% of overall diagnoses) and pheochromocytomas (21 cases, 29.3% of benign lesions and 22.3% of all cases). Other benign lesions included adrenal glands with fibrosis, and/or hemorrhage in 8.3% (six cases, 6.4% of all cases), adrenal cysts in 5.6% (four cases, 4.3% of total cases), and chronic inflammation in two cases. Solitary cases (1.4% of benign lesions, 1.1% of all lesions) of ganglioneuroblastoma, ganglioneuroma, lipoma, and schwannoma were found (Table 1).

**Table 1:** Distribution of histological categories in the benign lesions

**Histological category**	**Number of cases**	**Percentage of benign lesions**	**Percentage of total lesions**
Adenoma	31	43.1%	33.0%
Chronic inflammation	2	2.8%	2.1%
Fibrosis and hemorrhage	6	8.3%	6.4%
Ganglioneuroblastoma	1	1.4%	1.1%
Ganglioneuroma	1	1.4%	1.1%
Hyperplasia	4	5.6%	4.3%
Lipoma	1	1.4%	1.1%
Schwannoma	1	1.4%	1.1%
Pheochromocytoma	21	29.3%	22.3%
Cyst	4	5.6%	4.3%
Total	72	100%	76.6%

**Table 2:** Distribution of histological categories in the malignant lesions

**Histological category**	**Number of cases**	**Percentage of malignant lesions**	**Percentage of total lesions**
Primary lesions:			
Adrenocortical carcinoma	7	31.8%	7.4%
Pleomorphic leiomyosarcoma	1	4.5%	1.1%
Neuroblastoma	3	13.6%	3.2%
Metastatic lesions:			
Renal cell carcinoma	5	22.7%	5.3%
Colorectal adenocarcinoma	1	4.5%	1.1%
Gastroesophageal adenocarcinoma	1	4.5%	1.1%
Lung adenocarcinoma	2	9.1%	2.1%
B cell lymphoma	1	4.5%	1.1%
Urinary bladder carcinoma	1	4.5%	1.1%
Total	22	100%	23.4%

Metastases were the most common malignant lesion (50%) with renal cell carcinoma as the commonest site of origin (45.4% of metastatic and 22.7% of malignant lesions) followed by lung adenocarcinoma (9.1% of malignant lesions) and solitary cases (4.5% of malignant, 1.1% of total cases) of colorectal adenocarcinoma, gastroesophageal adenocarcinoma, B cell lymphoma and urinary bladder carcinoma. Primary malignant lesions included seven adrenocortical carcinomas (31.8% malignant lesions, 7.4% of all cases), three neuroblastomas (13.6% malignant and 3.2% total lesions) and one pleomorphic leiomyosarcoma (4.5% malignant and 1.1% total lesions) (Table 2).

### Uncommon adrenal incidentalomas

#### Case 1: adrenal ganglioneuroma

A 15-year-old female with idiopathic scoliosis underwent an MRI of the cervical, thoracic and lumbar spine to rule out neural axis abnormalities. An ovarian cyst was identified together with an incidental non-specific cystic structure. Kidneys were unremarkable on Doppler ultrasonography, and the non-specific cystic structure seen adjacent to the descending colon with an overall benign appearance was interpreted as a duplication cyst of the small bowel. At this stage, the initial MRI was reviewed. Retrospectively, on this review, a left adrenal mass was detected, measuring 3 × 2 × 4 cm. A high-resolution CT scan was ordered that showed washout properties inconsistent with a benign mass but without evidence of cystic change or hemorrhage. Differential radiological diagnosis included ganglioneuroma, ganglioneuroblastoma, neuroblastoma or pheochromocytoma. Clinical investigation included hormonal studies to evaluate catecholamine excess by the assessment of plasma metanephrines and urinary catecholamines and a metaiodobenzylguanidine (MIBG) scintiscan, which were negative, ruling out pheochromocytoma. Urinary free cortisol and demonstration of a normal diurinal rhythm of cortisol secretion by the administration of the dexamethasone suppression test were also negative ruling out subclinical cortical functional tumors. A laparoscopic left adrenalectomy was carried out. Her recovery was uneventful, and she was discharged 48 h later.

##### Pathology

**Figure 1:**
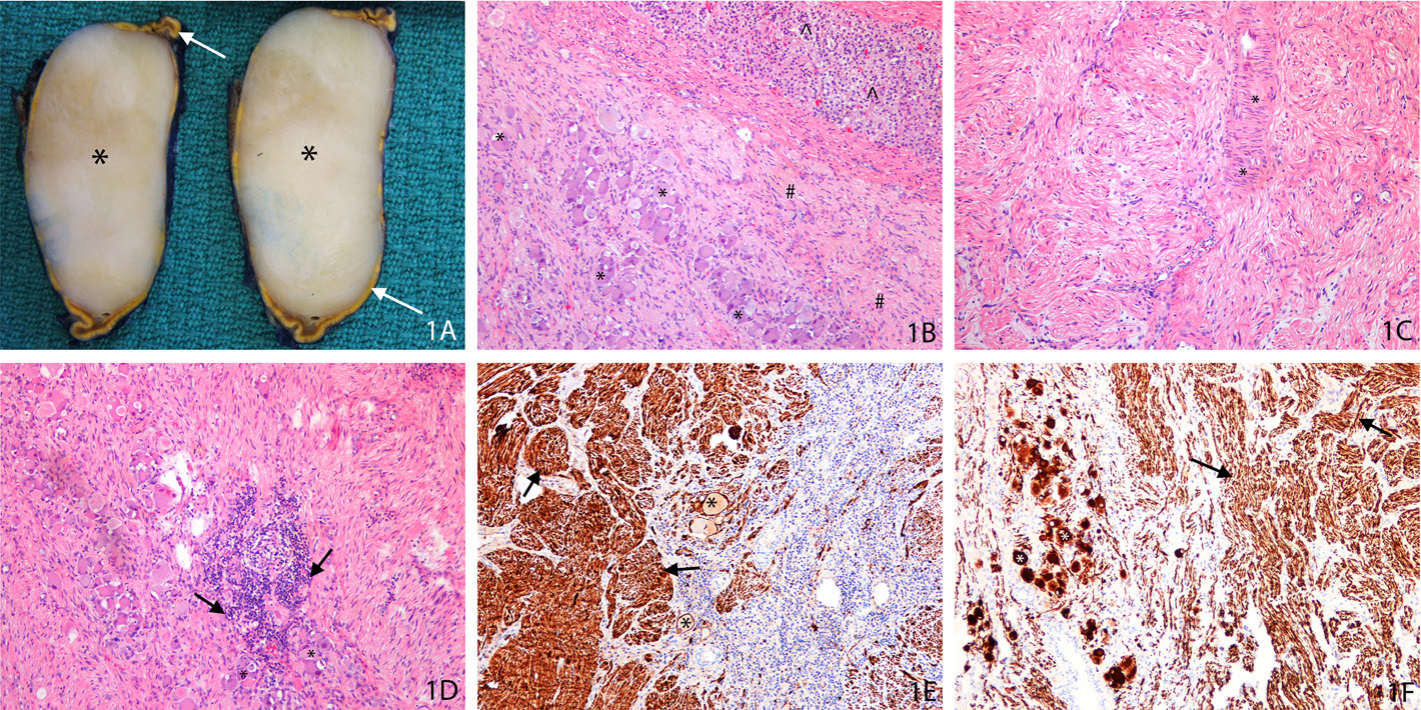
**Adrenal ganglioneuroma. (A)** Gross photograph of the cross section of the adrenal lesion shows a stretched rim of the residual, yellow colored adrenal (arrow) surrounding a well demarcated homogenous tan-colored lesion (*). **(B)** Photomicrograph of hematoxylin and eosin (H&E)-stained slide at low magnification shows expansion of the adrenal medulla by the lesional cells composed of mature ganglion cells (*) admixed with Schwannian-like spindle cells (#). The residual, uninvolved normal adrenal cortex is indicated (^). **(C)** Photomicrograph of H&E-stained slide at high magnification shows the Schwannian-like spindle cells with wavy benign nuclei and long stretched eosinophilic cytoplasm; * highlights a focus of vague nuclear pallisading. **(D)** Photomicrograph of H&E-stained slide at low magnification shows collections of benign lymphocytes (arrow) in a neurotropic distribution along the mature ganglion cells (*). **(E)** Photomicrograph of immunohistochemically-stained slide of S100 at low magnification shows the Schwannian-spindle cells (arrow) and the ganglion cells (*) to be strongly positive. **(F)** Photomicrograph of immunohistochemically-stained slide with neurofilament at low magnification shows the Schwannian-spindle cells (arrow) and the ganglion cells (*) to be strongly positive.

The left adrenal tumor was firm, tan, non-encapsulated and measured 3.5 × 2.2 × 2.4 cm in maximum dimension. On cross-sectioning, the mass was well demarcated with a stretched rim of normal-appearing adrenal tissue on either side (Figure 1A). On microscopic examination, the growth appeared to have expanded the medulla, causing compression of normal cortical and medullary tissue. The lesional cells were composed of Schwannian-like spindle cells with benign-looking nuclei and long stretched cytoplasm admixed with mature ganglion cells with well delineated cell borders, abundant eosinophilic cytoplasm and a large nucleus and nucleolus (Figure 1B,C). Some of the ganglion cells were multinucleated. Collections of benign lymphocytes were seen in neurotropic patterns of distribution along the clusters of the ganglion cells (Figure 1D). Scattered plasma cells with occasional mast cells were also seen. Evidence of hemorrhage, necrosis, mitotic activity and an immature embryonal component were not identified. Immunohistochemistry showed lesional cells to be strongly positive to S100 (Figure 1E), vimentin and neurofilament (Figure 1F). Though chromogranin was not expressed in the lesional cells, there was good internal control with positive staining in the medullary component of the residual adrenal gland. The final diagnosis was ganglioneuroma, with a Schwannian-stroma dominant mature subtype.

#### Case 2: periadrenal schwannoma

A 64-year-old non-smoker with uncontrolled hypertension (average 220/90 mmHg), poorly controlled type II diabetes, atrial fibrillation, depression and bilateral idiopathic blepharospasm underwent abdominal CT scanning for the investigation of abdominal wall swelling. A soft tissue mass on the medial aspect of the left adrenal gland measuring 3.1 × 2.7 × 4.1 cm was visualized incidentally. No additional mesenteric or retroperitoneal lymphadenopathy was identified, and the right adrenal gland, kidneys, spleen, and pancreas appeared normal. A renal Doppler ultrasound confirmed enlargement of the left adrenal gland, and further imaging with CT and MRI showed a solid enhancing mass with imaging features inconsistent with a benign adenoma. Based on his elevated blood pressure, and a 1-month history of biweekly episodes of sweating that lasted 5 minutes without associated chest pain, headache, shortness of breath or palpitations he was clinically suspected to have a pheochromocytoma. At two examinations, urine catecholamines were modestly elevated though metanephrines, vanillyl mandelic acid (VMA), homovanillic acid (HVA) and 5-hydroxyindoleacetic acid (5-HIAA) were normal on repeated testing. Due to the larger size of the tumor (4 to 4.5 cm), he underwent surgical exploration with a left laparoscopic radical adrenalectomy. Postoperatively his blood pressure remained stable and as there were no complications, the patient was discharged in 48 h.

##### Pathology

**Figure 2:**
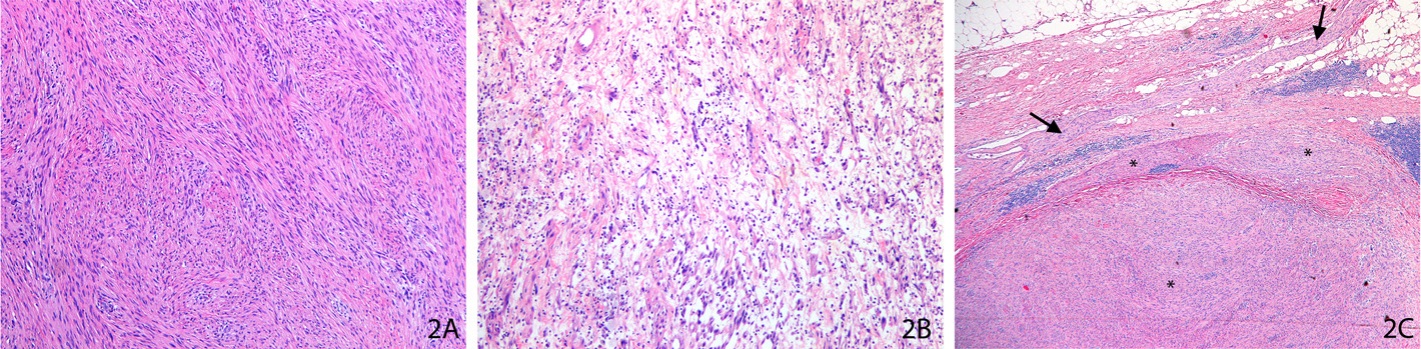
**Periadrenal schwannoma. (A)** Photomicrograph of hematoxylin and eosin (H&E)-stained slide at low magnification shows hypercellular Antoni A areas of spindle cells with abundant eosinophilic cytoplasm with wavy nuclei and vague pallisading. **(B)** Photomicrograph of H&E-stained slide at low magnification shows hypocellular Antoni B areas of scattered spindle cells in an abundant myxoid stroma. **(C)** Photomicrograph of H&E-stained slide at low magnification shows the well demarcated spindle cell neoplasm (*) arising from the nerve sheath of an adjacent periadrenal nerve fiber (arrow).

The specimen weighed 65 g and consisted of the left adrenal gland wrapped in mature adipose tissue. A large nodule was seen protruding adjacent to the adrenal gland, measuring 3.8 × 2.8 × 3.5 cm. Cut sections of the specimen revealed a well demarcated, fibrous mottled whitish-yellow lesion that grossly appeared to be directly adjacent to the adrenal gland with no direct involvement. On microscopic examination, the nodule was composed of (a) hypercellular areas of spindle cells with abundant eosinophilic cytoplasm, indistinguishable cell borders, and wavy nuclei with focal palisading areas (Figure 2A) and (b) hypocellular areas of spindle cells with stromal myxoid change (Figure 2B). This lesion was clearly demarcated from the adjacent adrenal gland, which was intact and unremarkable. The lesion appeared to arise from an adjacent periadrenal retroperitoneal nerve sheath fiber as depicted in Figure 2C. Immunohistochemistry showed the lesional spindle cells to be strongly positive to S100, confirming their neural lineage. The histomorphological features combined with the immunophenotype confirmed the diagnosis of a periadrenal schwannoma with hypercellular Antoni A and hypocellular Antoni B areas.

#### Case 3: primary adrenal pleomorphic leiomyosarcoma

A 28-year-old female who smoked half a pack of cigarettes a day with no significant medical history was being investigated for abdominal pain. A CT scan was ordered, which revealed a large left adrenal mass adherent to the kidney. Preoperative investigations including hormonal studies to evaluate catecholamine excess by the assessment of plasma metanephrines, urinary catecholamines and a MIBG scintiscan were negative, ruling out pheochromocytoma. Urinary free cortisol and demonstration of a normal diurinal rhythm of cortisol secretion by the administration of the dexamethasone suppression test were also negative ruling out subclinical cortical functional tumors. The larger size of the mass was highly suspicious for malignancy; therefore an open surgical intervention was planned. At surgery, via a left thoracoabdominal incision an en-bloc resection of the left adrenal mass was carried out including left nephrectomy, partial diaphragmatic resection with reconstruction and placement of a left thoracostomy. Postoperatively, the only significant complication was an asymptomatic left-sided pneumothorax that resolved spontaneously upon removal of the chest tube. She was discharged with an outpatient referral to the Cancer Center in 6 weeks. No further postoperative information is available for review in the current health record system.

##### Pathology

**Figure 3:**
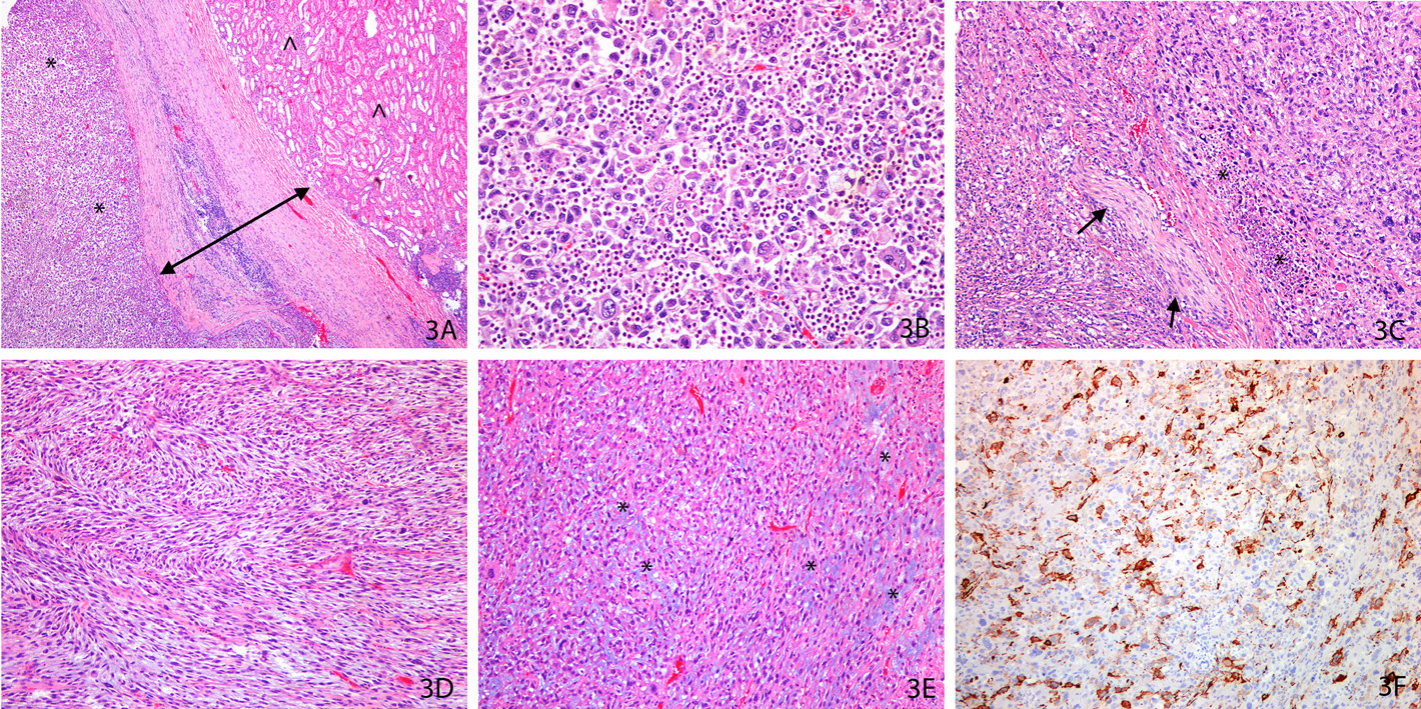
**Primary adrenal pleomorphic leiomyosarcoma. (A)** Photomicrograph of hematoxylin and eosin (H&E)-stained slide at low magnification shows a well demarcated neoplastic lesion (*) separated by a pseudocapsule (arrows) from the adjacent normal, uninvolved renal parenchyma (^). **(B)** Photomicrograph of H&E-stained slide at high magnification shows the tumor cells to be composed of sheaths of pleomorphic cells with marked cytological atypia including large bizarre cells with irregular nuclei and prominent nucleoli. **(C)** Photomicrograph of H&E-stained slide at moderate magnification shows areas of focal necrosis (*) and perineural invasion (arrow). **(D)** Photomicrograph of H&E-stained slide at moderate magnification shows sheets of neoplastic spindle cells in a prominent storiform pattern. **(E)** Photomicrograph of H&E-stained slide at moderate magnification demonstrates heterogeneity of the tumor cells in a marked myxoid (*) background. **(F)** Photomicrograph of immunohistochemically-stained slide with smooth muscle actin (SMA) at low magnification shows strong expression within the lesional cells.

The gross specimen weighing 1,492 g consisted of the left kidney and adrenal gland, with portions of the diaphragm. A large 16.5 × 13.5 × 12.5 cm ragged, irregular, pale-tan rubbery multinodular mass with hemorrhage and necrosis was seen completely replacing the left adrenal gland with no residual normal tissue being identified. On microscopic examination, the tumor was surrounded by a pseudocapsule distinct from the adjacent renal parenchyma (Figure 3A) and composed of groups of pleomorphic cells (Figure 3B) with areas of necrosis, hemorrhage and perineural invasion (Figure 3C). Heterogeneity of the tumor was seen including tumor cells being arranged in sheets and storiform patterns with extensive fibrous spindled stroma in some areas (Figure 3D) and others with a myxoid background (Figure 3E). Proliferating cells varied from bland to highly pleomorphic with histiocytoid appearance to multinucleated giant cells with a wide range of cytological atypia including large bizarre cells, irregular nuclear contours, multiple prominent eosinophilic nucleoli, nuclear grooves and folds, rare atypical mitoses, and hyperchromatic cells with eosinophilic cytoplasm (Figure 3B). Typical features of benign smooth muscle tumors such as bland spindled neoplastic cells with cigar-shaped nuclei arranged in fascicles were not observed. The tumor was well demarcated from the adjacent renal parenchyma by a thick fibrous pseudocapsule as seen in Figure 3A.

Immunohistochemical examination showed the neoplastic cells to be strongly positive for vimentin and smooth muscle actin (SMA) (Figure 3F) with negative staining to Pan-Keratin, cytokeratin (CK)7, CK20 and high-molecular-weight keratin (HMWK) confirming a sarcomatous lineage. Rhabdomyosarcoma was excluded by negative staining to myogenin and desmin. S100 and other melanoma markers were also negative. P53 and Ki67 were moderately expressed in the lesional cells. *In situ* hybridization for Epstein-Barr virus was negative. A diagnosis of primary pleomorphic leiomyosarcoma was suggested due to the cells being strongly positive to SMA. The final diagnosis of high-grade primary pleomorphic leiomyosarcoma of the adrenal gland was confirmed with corroborative external consultation due to the rarity of this lesion.

## Discussion

**Figure 4:**
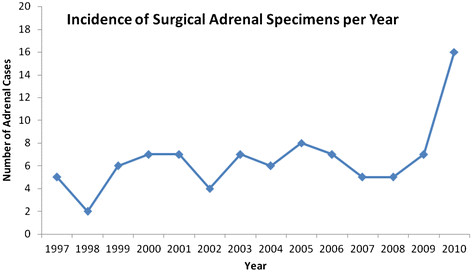
**Incidence of surgical adrenal specimens per year.** This line graph illustrates the total number of adrenal specimens received in the surgical pathology laboratory between 1997 and 2010. The x-axis records the years in a consecutive chronological order and the y-axis records the number of cases (0 to 20) in intervals of 2.

Improvements in non-invasive sophisticated abdominal imaging techniques coupled with their growing popularity and acceptance have resulted in the increasing detection of clinically silent adrenal lesions, ‘incidentalomas’ [5]. Such trends are reflected in our study, as seen in Figure 4 that illustrates a dramatic spike of surgical adrenal specimens received in 2009 to 2010. These ‘incidentalomas’ are adrenal masses over 1 cm in diameter found serendipitously during radiographic imaging for non-adrenal related investigations as seen in our index cases [6,7]. Once discovered, such lesions require diagnostic evaluation to determine whether they are hormonally functional vs non-functional, and/or malignant vs benign [8]. The prevalence of incidentalomas increases from 2.1% in the general population to 7% in patients older than 70 years [9]. The most common incidentaloma is an adrenal cortical adenoma, accounting for 36% to 94% of cases [3]; however, any tumor type, benign or malignant, may present serendipitously [6]. Incidentalomas may be (a) medullary based, including pheochromocytoma, ganglioneuroblastoma, neuroblastoma or ganglioneuroma or (b) cortical based, including adenoma, myelolipoma, or adrenocortical carcinoma [1]. These tumors are most commonly non-functional lesions (85%) [8]; however, up to 20% of patients will demonstrate some form of subclinical hormonal dysfunction [2] with 9% of patients having subclinical Cushing’s syndrome, and 1% with aldosteronomas [8]. Excessive cortisol or aldosterone secretion is more often associated with benign adenomas, whereas sex steroid secretion may be indicative of a malignant neoplasm [1]. Consistent with the results seen in our surgical pathology review (Tables 1 and 2), benign, non-functioning adrenal cortical adenomas are the most common adrenal incidentalomas (33.0%) while metastatic disease has a moderate frequency (11.8%). The latter is comparatively high in relation to the literature wherein only 1 in 4,000 adrenal tumors are reported as malignant [1,2] with the majority of these being metastatic. We believe that this increased percentage of malignant pathologies seen in our series is directly related to the selection criteria of this review that is limited to adrenal incidentalomas that have been subjected to surgical intervention resulting in surgical specimens that are available for pathological examination. In 75% of patients with a history of malignant disease, primary tumor sites that often metastasize to the adrenal originate from the lung, kidney, colon, breast, esophagus, pancreas, liver, and stomach. These adrenal deposits will often be bilateral. The risk of malignancy in an adrenal mass is usually estimated based on its presentation, hormonal status, radiological size, and histological features on biopsy [1].

An optimal diagnostic approach for the management of adrenal incidentalomas has yet to be determined; however, certain recommendations have been proposed [1,2,10]. A 24-h urine specimen to measure fractionated metanephrines and catecholamines is recommended for all patients with adrenal incidentalomas, as raised metanephrine and/or catecholamine levels may suggest pheochromocytoma with a sensitivity of 91% and specificity of 98% [10]. Early characterization of this diagnosis is important, as it is critical that it be firmly excluded in order to carry out a fine-needle aspiration biopsy (FNAB) due to the high risk of hypertensive crisis in a catecholamine-secreting tumor [2,11]. FNAB does not exclude malignancy due to the high false negative rate, with up to 20% of such specimens being insufficient for diagnosis [12]. Complications including adrenal hematoma, abdominal pain, hematuria, pancreatitis, pneumothorax, formation of an adrenal abscess and tumor recurrence along the needle tract may also be encountered in 5% to 8% [10]. Adrenocortical scintigraphy provides both anatomical localization and *in vivo* functional characterization of the adrenal glands due to the uptake and accumulation of the radio tracer. In this context, MIBG and N-59 scans for functioning adrenal medullary and cortical tissues, respectively, are undertaken to rule out any subclinical medullary/cortical functional tumors.

Preoperative radiological imaging including CT scans and MRI are used to determine size of the adrenal tumor, which dictates the need for surgical intervention and influences the surgical approach employed by the surgeon. However, as seen in the study by Kouriefs *et al*., preoperative CT and MRI tend to significantly underestimate the actual histological size of adrenal tumors larger than 3 cm by 18.1% and 20%, respectively. In this context, it is recommended that three-dimensional reconstruction of spiral CT or MRI images may overcome this problem of size underestimation [4,13]. CT imaging criteria of an unenhanced CT attenuation coefficient of 0 Hounsfield units (HU) or less has been reported to be 100% specific in the distinction of a benign adenoma from a metastatic lesion; however, many recently reported studies show discrepant results with thresholds of 10 HU and 16.6 to 18 HU yielding sensitivities of 58% and 88% to 100% and specificities of 92% and 95% to 100%, respectively [4]. Similarly, MRI imaging criteria including signal intensity ratios on T1-weighted and T2-weighted sequences do not seem to consistently help in distinguishing benign adrenal lesions from malignancies [4]. Additionally, the size of the tumor by MRI is underestimated by 20% in comparison to 16% to 47% by CT scans [14]. The size of the tumor however remains the key determinant of prognosis, and dictates patient management. Statistically, of tumors less than 4 cm in diameter, over 60% are benign adenomas, while less than 2% are adrenocortical carcinomas with the risk of adrenocortical carcinoma increasing by 25% in lesions larger than 6 cm [8]. However, there is a growing consensus that adrenal tumor size should not be used to establish malignant vs benign diagnosis [13]. Such findings have prompted the recommendation of laparoscopic adrenalectomy for incidentalomas less than 4 cm with a radical open surgical excision for those over 6 cm. Uncertainty regarding management remains for all adrenal lesions that fall between these size limits (4 to 6 cm) [15]. Factors including the patient’s age and health status as well as the mass’s imaging phenotype should be considered in the management planning of these cases [10]. These parameters for laparoscopic resection may not be valid in the pediatric population, as tumor size must be considered in relation to the child’s size [16]. The first laparoscopic adrenalectomy was reported over 25 years ago (1992) and has since been adopted as the primary treatment modality for many adrenal lesions [16]. This procedure offers the advantages of less postoperative discomfort, a shorter hospital stay, fewer postoperative disabilities, and a lower rate of complications [14].

Cystic masses in the adrenal are rare and documented incidence varies between 0.064% to 0.18% in autopsy series [17,18]. Four adrenal cysts were seen in our series, with three being endothelial-lined and one epithelial. Adrenal cystic lesions are usually incidental and asymptomatic. Histologically, adrenal cysts are classified into four major types: (a) endothelial cysts, (b) pseudocysts, (c) parasitic cysts and (d) epithelial cysts. Endothelial cysts are the most common pathological subtype seen, representing 45% of adrenal cysts with pseudocysts being the second most common (39%). Epithelial and parasitic cysts are extremely rare [17,19]. Solid masses in the adrenal include commonly encountered lesions such as adenoma and pheochromocytoma with uncommon lesions such as ganglioneuroma, ganglioneuroblastoma, extramedullary plasmacytoma, primary lymphoma, schwannoma, myelolipoma, mature teratoma, and tuberculoma.

The following section focuses on the discussion of three rare lesions encountered as adrenal incidentalomas in our surgical pathology practice.

### Adrenal ganglioneuroma

Ganglioneuromas are benign, slow-growing neoplasms that arise from primordial neural crest tissue containing mature Schwann cells, ganglion cells, and nerve fibers [12]. These tumors are most common in the retroperitoneum (35% to 52%), mediastinum (39% to 43%) or cervical region (8% to 9%). The adrenal gland is a rare location for ganglioneuromas to originate [20]. Ganglioneuromas of adrenal origin are more common in the younger population in some studies [12]; however, other studies report the mean age at diagnosis to be usually between the fourth and fifth decades of life [21]. In our index case, the patient was 15 years old. Ganglioneuromas are uncommon in the adrenal gland, making up 0% to 6% of all incidentalomas [14]. The true incidence of these lesions however cannot be determined as adrenal ganglioneuromas are generally asymptomatic and therefore often undetected. Up to 37% of tumors can produce catecholamines or other hormones, with hormonally-induced paraneoplastic syndromes such as diarrhea and hypertension from increased vasoactive intestinal peptide (VIP) or virilization from testosterone secretion [21,22].

MRI and CT features vary between tumors due to the mixed composition of ganglion and Schwann cells [23]. In this context, it can be challenging to accurately diagnose ganglioneuromas preoperatively [20]. A recent study found that approximately 65% of adrenal ganglioneuromas were misdiagnosed preoperatively [12] and based on imaging were commonly interpreted as being malignant [23]. Ultrasonography detects a homogeneous, hypoechoic mass with well defined borders; however, usually the quality of the mass cannot be assessed with certainty [15,20]. CT scan images usually show a well circumscribed low-attenuated homogeneous mass [24]. Microcalcification or macrocalcification is detected in 20% to 60% of ganglioneuromas [23]. On MRI imaging, ganglioneuromas have a low signal intensity on T1-weighted images (T1WI) and heterogeneous, high intensity on T2-weighted images (T2WI) [17]. These findings vary however with tumor content, as high myxoid stroma and low collagen yields a higher T2WI signal intensity while the whorled appearance on MRI is variable and correlates with the presence of Schwann cells and collagen fibers [23].

Definitive diagnosis of ganglioneuroma of the adrenal gland is only by microscopic examination. Ganglioneuromas are encapsulated on gross examination, with a solid, homogeneous gray-white cut surface as seen in Figure 1A in our reported case. They can be up to 18 cm in size and their consistency may range from gelatinous to solid [21]. Microscopically, areas of Schwann cells with wavy dark nuclei and inconspicuous nucleoli with scant spindled cytoplasm are seen admixed with mature ganglion cells and eosinophilic cytoplasm, large vesicular nuclei and prominent nucleoli [21]. Immunohistochemical staining of these two cell types can aid in the diagnosis of ganglioneuroma, with the Schwann cells being strongly positive for S100 and the ganglion cells being immunoreactive to synaptophysin, CD56, neuron-specific enolase, and neurofilament proteins as seen in Figure 1D of our case. Uncertainty of preoperative diagnosis usually results in adrenal ganglioneuromas being treated surgically with a view to complete operative resection as carried out for adrenal carcinomas [24]. Ganglioneuroma has an excellent prognosis, and recurrences are rare after surgical resection [14].

### Adrenal/periadrenal schwannoma

Schwannomas are slow-growing benign nerve sheath tumors that originate from neural crest cells and most commonly arise in the head, neck, upper and lower extremities, and trunk [5,25]. Rare visceral sites of involvement include the gastrointestinal tract, liver, pancreas, kidney, brain, heart, and retroperitoneum [6,25]. Of all retroperitoneal masses, schwannomas account for only 1% to 5% [26]. Only 0.7% of benign and 1.7% of malignant schwannomas are found in the retroperitoneum [27]. Schwannomas of the retroperitoneum usually present with abdominal or back pain [26]; however, many different types of symptoms have been described and in such cases diagnosis is only made intraoperatively or postoperatively. Periadrenal schwannoma mimicking an adrenal mass as seen in our reported case, though uncommon, has previously been reported in the literature [5].

Schwannomas arising specifically within the adrenal gland are extremely rare [28,29]. It is believed that these adrenal lesions originate from the Schwann cells of nerve fibers innervating the medulla [30]. These include a sympathetic branch from the upper lumbar plexus, the phrenic, or the vagus nerves. Schwannoma has not been identified arising from the cortex, presumably because this region contains only a few thin nerves running along its vasculature [29]. Usually clinically non-secreting and asymptomatic, adrenal schwannoma is often an incidental finding [25]. Mass effect may, however, result in upper abdominal and/or flank pain [30]. CT scan shows a well circumscribed, homogeneous, round/oval mass that may show cystic degeneration or calcification [25]. MRI shows solid tumors with a low-signal intensity on T1WI and heterogeneously high intensity on T2WI [29]. In our reported case, the patient’s significant hypertension, elevated catecholamines, and the location/characteristics of the lesion on imaging were all highly suspicious for a malignant adrenal neoplasm. Heterogeneity and degeneration in some retroperitoneal schwannomas may mimic malignancy on radiological interpretation [31]. Preoperative diagnosis of adrenal vs periadrenal schwannoma is almost impossible and usually confirmed only on histopathological examination as seen in our reported case.

On gross examination adrenal/periadrenal schwannomas often appear as a solitary, well circumscribed tumor enveloped in a fibrous capsule with a solid firm white-tan and homogeneous cut surface that may contain cystic changes or hemorrhagic regions [6,32]. Periadrenal/adrenal schwannomas are microscopically identical to schwannomas found at any other anatomical sites [30]. Spindle cells are visualized with alternating areas of compact hypercellularity with irregular streams and elongated spindle cells (Antoni type A) and of loosely textured hypocellularity with cystic spaces (Antoni type B) [28,32,33]. Usually one Antoni type is predominant [25]. Verocay bodies between regions of nuclear palisades and thick-walled hyalinized blood vessels may additionally be seen [28,29]. Immunohistochemically, these lesions stain positively for S100 antigen, collagen IV and laminin, and negatively for keratin, desmin, actin, and CD34. Laparoscopic adrenalectomy is the mainstay treatment for periadrenal/adrenal schwannomas, and postoperative recovery is usually uneventful [33]

### Primary pleomorphic adrenal leiomyosarcoma

Leiomyosarcoma is a mesenchymal malignancy of the smooth muscle that most commonly arises in the myometrium, retroperitoneum, and the respiratory tract [34]. Primary mesenchymal adrenal tumors are rare, and more often adrenal leiomyosarcoma is metastatic or an extension from the retroperitoneum [35]. Only 18 cases of primary adrenal leiomyosarcoma were reported in the literature in 2009 [36]. The source of these rare tumors remains unknown, with origin being suggested from the smooth muscle layer of the adrenal blood vessel [37]. Patient ages ranged from 30 to 68 years, with no laterality or sex predilection [38]. Typically patients are older and present with large tumors [34]. Most adrenal leiomyosarcomas are larger than 10 cm in diameter. On imaging studies, adrenal leiomyosarcoma is indistinguishable from adrenocortical carcinoma and metastatic cancers [38]. As they are generally clinically asymptomatic, discovered as incidentalomas as seen in our reported case, early detection of this malignancy is further hampered by a lack of applicable tumor markers and its rapid growth [34,38].

The exact etiology of these neoplasms remains unclear. Immunocompromised patients including transplant recipients, those on chemotherapy and those with HIV/AIDS are predisposed to the development of smooth muscle tumors (SMT). They are also reported to have an increased risk for developing adrenal leiomyosarcoma [36]. In this context, Epstein-Barr virus infection has been reported to be involved in the pathobiogenesis of these SMTs [39,40]. Our index patient, though young (28 years), was immunocompetent (HIV negative) and showed no evidence of Epstein-Barr virus (EBV) by *in situ* hybridization of the tumor.

Optimal management of this rare lesion recommends complete resection with a minimum of 3 cm margins, though this is often not feasible due to local invasion [41]. Venous thrombosis, adjacent organ invasion and distant metastases are correlated with an extremely poor prognosis [38]. Some reports suggest a role for postoperative adjuvant therapy including chemotherapy and radiotherapy as being beneficial in controlling local disease. On microscopic evaluation, an adrenal mass demonstrating malignant spindle and bizarre pleomorphic cells should raise the suspicion of leiomyosarcoma, either primary or metastatic, the latter being more common [34].

Primary adrenal pleomorphic leiomyosarcoma is an extremely rare lesion, with only three cases reported in the English literature as listed in Table 3 (PubMed and Medline) [34,42,43]. More females are reported with this variety of leiomyosarcoma than males, and the left adrenal gland is more commonly affected (Table 3). Due to the paucity of cases, the diagnostic criteria and biological behavior of this lesion remain controversial [43]. On histological examination, this variant has pleomorphic areas similar to storiform/pleomorphic malignant fibrous histiocytoma and histological differentiation can be extremely difficult. As such, extensive sampling to detect foci of low-grade leiomyosarcoma as well as detailed immunostaining is recommended. Osteoclast-like giant cells have been described in these tumors [43]. A panel of immunohistochemical stains is often required to definitively arrive at this diagnosis, which is frequently made by exclusion of other high-grade malignant mimics such as malignant melanoma, poorly differentiated carcinoma, and/or high-grade undifferentiated sarcomas. Other diseases that must be effectively ruled out include metastatic carcinoma, sarcomatoid renal cell carcinoma, adrenocortical carcinoma, metastatic sarcoma, retroperitoneal sarcoma, malignant fibrous histiocytoma, epithelioid angiosarcoma and pleomorphic rhabdomyosarcoma. In all cases, a metastatic source of leiomyosarcoma should be excluded. These tumors are heterogeneous and demonstrate variable expressions of smooth muscle actin, muscle-specific actin, and desmin in different parts of the tumor [34]. Due to the limited number of cases, the long-term behavior of this lesion remains anecdotal; however, like adrenocortical carcinoma, organ-confined lesions have improved prognoses. Postoperative adjuvant radiation therapy is recommended in locally advanced malignancies. The role of chemotherapy is not well documented due to the rarity of this lesion.

**Table 3:** Adrenal pleomorphic leiomyosarcoma (PubMed and Medline search ‘pleormorphic leiomyosarcoma’ AND ‘adrenal’, limited to English language)

**Ref no.**	**First author and year**	**Age (sex)**	**Presenting symptoms**	**Laterality**	**Primary treatment**	**Metastases**	**Outcome**
This work	Kanthan R, 2011	28 (F)	None (incidentaloma)	Left	Adrenal resection, nephrectomy, diaphragm resection and reconstruction	Unknown	Unknown
32	Mohanty SK, 2007	47 (F)	Abdominal pain, nausea, vomiting, deep vein thrombosis	Left	Adrenalectomy, nephrectomy, radiotherapy	Bilobar hepatic and bilateral pulmonary nodules, left hilar lymph node at 9 months	Treated with combination chemotherapy and close follow-up
39	Candanedo-González FA, 2005	59 (F)	Abdominal pain, 4 kg weight loss/3 months	Left	Laparotomy with adrenalectomy	Local recurrence and liver metastases at 12 months	Adjuvant chemotherapy and radiotherapy with metastasectomy; alive 24 months later, no evidence of disease
38	Lujan MG, 2002	63 (M)	1-year history of enlarging abdominal mass	Right	Preoperative chemotherapy, cholecystectomy, right hepatic lobectomy, right adrenalectomy	Pulmonary, hepatic metastases and advanced local disease at time of surgery	Death shortly after surgery

## Conclusions

Adrenal lesions are rare specimens in the surgical pathology laboratory. With increased and improved use of sophisticated diagnostic imaging techniques it is predicted that the number of adrenal incidentalomas detected will continue to rise. Laparoscopic or open surgical exploration of such lesions will result in increased specimens for pathological analysis. In this context, awareness of common and uncommon benign and malignant lesions of the adrenal medulla and cortex is vital for accurate pathological diagnosis to guide optimal patient management.

## Competing interests

The authors of this article have no relevant financial relationships with commercial interests to disclose.

## Authors’ contributions

All authors participated in the creation and revisions of this manuscript. All authors read and approved the final manuscript.
